# Patpat: a public proteomics dataset search framework

**DOI:** 10.1093/bioinformatics/btad076

**Published:** 2023-02-06

**Authors:** Weiheng Liao, Xuelian Zhang

**Affiliations:** State Key Laboratory of Genetic Engineering, School of Life Sciences, Fudan University, Shanghai 200438, China; State Key Laboratory of Genetic Engineering, School of Life Sciences, Fudan University, Shanghai 200438, China

## Abstract

**Summary:**

As the FAIR (Findable, Accessible, Interoperable, Reusable) principles have become widely accepted in the proteomics field, under the guidance of ProteomeXchange and The Human Proteome Organization Proteomics Standards Initiative, proteomics public databases have been providing Application Programming Interfaces for programmatic access. Based on generating logic from proteomics data, we present Patpat, an extensible framework for searching public datasets, merging results from multiple databases to help researchers find their proteins of interest in the vast mass spectrometry. Patpat’s 2D strategy of combining results from multiple databases allows users to provide only protein identifiers to obtain metadata for relevant datasets, improving the ‘Findable’ of proteomics data.

**Availability and implementation:**

The Patpat framework is released under the Apache 2.0 license open source, and the source code is stored on GitHub (https://github.com/henry-leo/Patpat) and is freely available.

**Supplementary information:**

Supplementary data are available at *Bioinformatics* online.

## 1 Introduction

Hypothesis derivation is a common approach in modern scientific research. When a researcher is interested in a protein of unknown function, he may try to find out how this protein is expressed in various physiological states, thus generating a hypothesis about the function, and proteomic data provide the conditions conducive to the formation of the hypothesis. Guided by the FAIR (Findable, Accessible, Interoperable, Reusable) principles (Wilkinson *et al.*, 2016) and HUPO-PSI (Deutsch *et al.*, 2017), a series of proteomic data submission and distribution standards have been established. With the support from PX Consortium (Deutsch *et al.*, 2020) members, the data become increasingly standardized and facilitate researchers to share their research results. With the advent of big data in proteomics, how to better reuse proteomic data to drive scientific progress has become a popular research topic.

**Fig. 1.**
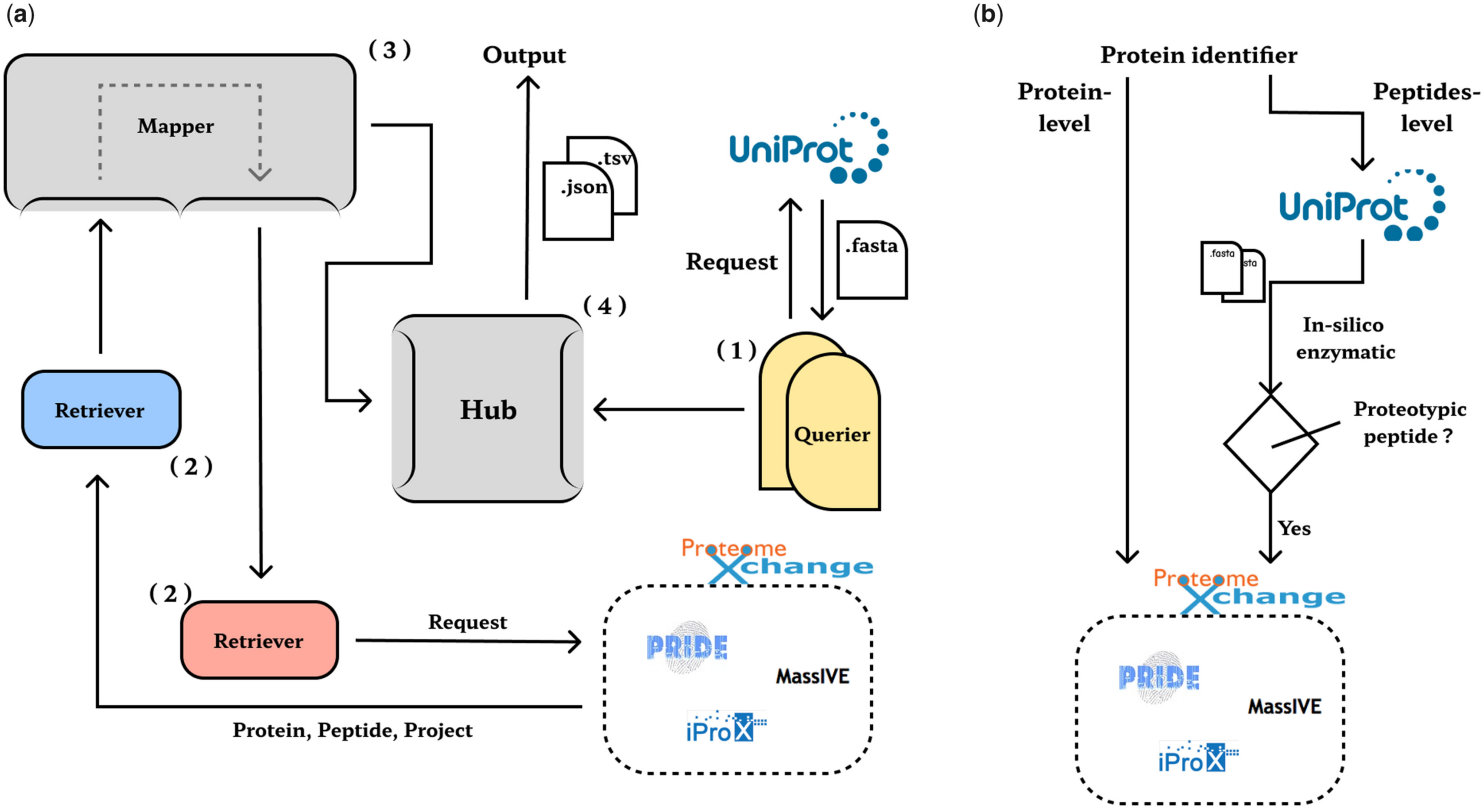
(**a**) Patpat as a whole is designed using the builder model, with each module implementing different functions that work together to keep Patpat running correctly; (1) Querier: to implement protein metadata queries and the generation of peptide; (2) Retriever: to implement direct interaction with the public database; (3) Mapper: to arrange the Retrievers and implement the dataset mapping; (4) Hub: to integrate the information and export the results. (**b**) 2D search strategy. It interacts with public databases via protein identifiers and unique peptides, respectively

To help researchers find datasets of interest in the vast amount of proteomics data available, we present Proteomics Aiders Telescope (Patpat), a Python-based framework for searching public proteomics datasets, with which users can search for metadata of relevant datasets with just a few lines of code. In addition, Patpat is an extensible and pluggable search framework. On the one hand, Patpat is designed by using the builder pattern, where each module performs a specific function and is integrated by a unified Hub module (Fig. 1a). In contrast, the internal design of each module uses the factory pattern, which only requires the inheritance of base classes to extend the module’s functionality, making it easy for developers to integrate their databases of interest into the framework or build personalized search solutions. On the other hand, Patpat can merge search results from multiple databases, providing researchers with a one-stop search experience. Patpat supports PRIDE (Perez-Riverol *et al.*, 2022), iProX (Ma *et al.*, 2019) and MassIVE (Doerr, 2019) databases. See the Wiki for more information.

Eukaryotes have evolved a series of error correction mechanisms for transcriptional processes over a long period to ensure accurate gene expression. The human protein Regulator of nonsense transcripts 2 (UPF2) has an essential function as a binding protein involved in nonsense-mediated mRNA decay (Lykke-Andersen *et al.*, 2000). In this article, we search for UPF2 and compare it with other mainstream search tools to demonstrate the usability and specificity of Patpat.

## 2 Materials and methods

The ‘bottom-up’ strategy is a ‘peptide-centric’ detection strategy, where peptide sequences are inferred from Mass Spectrum, and proteins are identified against a given protein sequence database. The ‘bottom-up’ strategy is widely used in proteomics (Zhang *et al.*, 2013). For protein identification, the unique peptide is often the best evidence for the presence of a protein (Nesvizhskii and Aebersold, 2005). Based on the logic of ‘bottom-up’ proteomics data generation, Patpat uses a 2D (protein-level and peptides-level) strategy to search public databases and map the results to datasets (Fig. 1b).

We take the search for the human protein UPF2 as an example. First, the UniProt identifier Q9HAU5 of UPF2 is provided to Patpat, which interacts with the UniProt database (UniProt Consortium, 2021) to obtain the sequence and proteomic information of the protein. After that, the target protein and the proteomic library it belongs to is *in silico* enzymatic digestion with the same parameters. Here, we use the trypsin digestion rules and allow one missed cleavage, peptide lengths from 7 to 25. If a peptide is derived from the target protein only, it is a unique peptide. After the queries, the protein has 163 unique peptides (Supplementary Data), and the sequence information is saved to the search config. In the process, Patpat obtains the proteomic library from UniProt by default, or the user can download it locally. The Querier module performs this process, and the *in silico* enzymatic digestion method is supported by the Pyteomics framework (Levitsky *et al.*, 2019).

Secondly, Patpat interacts with the public database API based on user config via protein identifiers and unique peptide sequences, respectively and completes the mapping of the dataset by retrieving the results (The design of the PRIDE and iProX APIs is attached here). Note that the type of retrieval results is specified by the database API and is not controlled by Patpat. Here, we have selected the PRIDE and iProX databases to search. The retrieval results are stored as the Universal Spectrum Identifier (USI). USI is PSI published spectral distribution standard (Deutsch *et al.*, 2021) that advances the proteomic data distribution standard from mass spectrometry file identification to spectral evidence granularity, promoting the spectral evidence ‘FAIRness’. Based on the USI format design, the ‘Collection Component’ is the dataset identifier through which Patpat can obtain information about the dataset to be retrieved and complete the mapping. The Mapper module and the Retriever module support the retrieval and mapping process.

Finally, Patpat filters the dataset based on information about the organism to which the protein belongs to ensure that the peptides retrieved in each dataset are evidence of the presence of the target protein. The filtered search results are delivered to the user in JSON and TSV files. The JSON file stores the complete information of the filtered dataset, the information richness of which depends on the metadata in the corresponding database. The TSV stores the standardized information of the dataset, including title, summary and project URL information. See the Wiki for details on how to use the search results.

## 3 Results and discussion

Based on mapping and filtering (2022-09-22), 273 UPF2-related datasets have been searched (Supplementary Data). We randomly selected two datasets, PXD014753 and PXD001985, from the search results and selected five (if existing) peptides-level search results, respectively, to check the corresponding protein identification in the public database (Supplementary Data), and found that among them, UPF2 was identified with RENT2_HUMAN as the number, instead of UPF2 or Q9HAU5, which was determined by the proteomic sequence library used in the spectral identification process. The peptides-level search solves exactly this problem.

In addition, most of the search results come from searches at the peptides level, and searches at the peptides level also cover most searches at the protein level (Supplementary Fig. S1). With only two datasets obtained separately for the protein level, a simple API query cannot yield such results, showing the benefits of Patpat searches. Upon inspection, the search results for these two datasets (PXD002471: LVKETTVKSLDK; PXD007632: KEEKTPNITK, LVKETTVK) were not in the list of unique peptides generated by Patpat (Supplementary Data), i.e. these two proteins were identified by non-unique peptides.

Further, we compared Patpat’s search results with those of OmicsDI (Perez-Riverol *et al.*, 2017) (Supplementary Fig. S2). In terms of the number of results, Patpat is less than OmicsDI. However, although both OmicsDI and Patpat support PRIDE and iProX databases, OmicsDI searches do not contain results from PRIDE and iProX but from PeptideAtlas (van Wijk *et al.*, 2021). In contrast, Patpat finds 273 datasets in these two databases, showing Patpat’s data discovery capabilities.

In conclusion, as a dataset search framework more in accord with proteomics, Patpat has some application value for mining dataset metadata of interest to researchers from massive databases. It is also worth mentioning that Patpat, as a database-free API access tool, does not store data itself. While it can circumvent specific copyright issues, it has some drawbacks regarding search speed or external server downtime compared to database-based search tools.

In addition, the Patpat progress bar is supported by the tqdm package (da Costa-Luis *et al.*, 2019), and the diagram used in this article was created by Figma software.

## Supplementary Material

btad076_Supplementary_Data

## Data Availability

The data underlying this article are available in the article and in its online supplementary material.
