# Supplementary figures and images for: Patpat: a public proteomics dataset search framework
Source: Bioinformatics. 2023 Feb 6;39(2):btad076. doi: 10.1093/bioinformatics/btad076 (PMC9933831; doi:10.1093/bioinformatics/btad076)

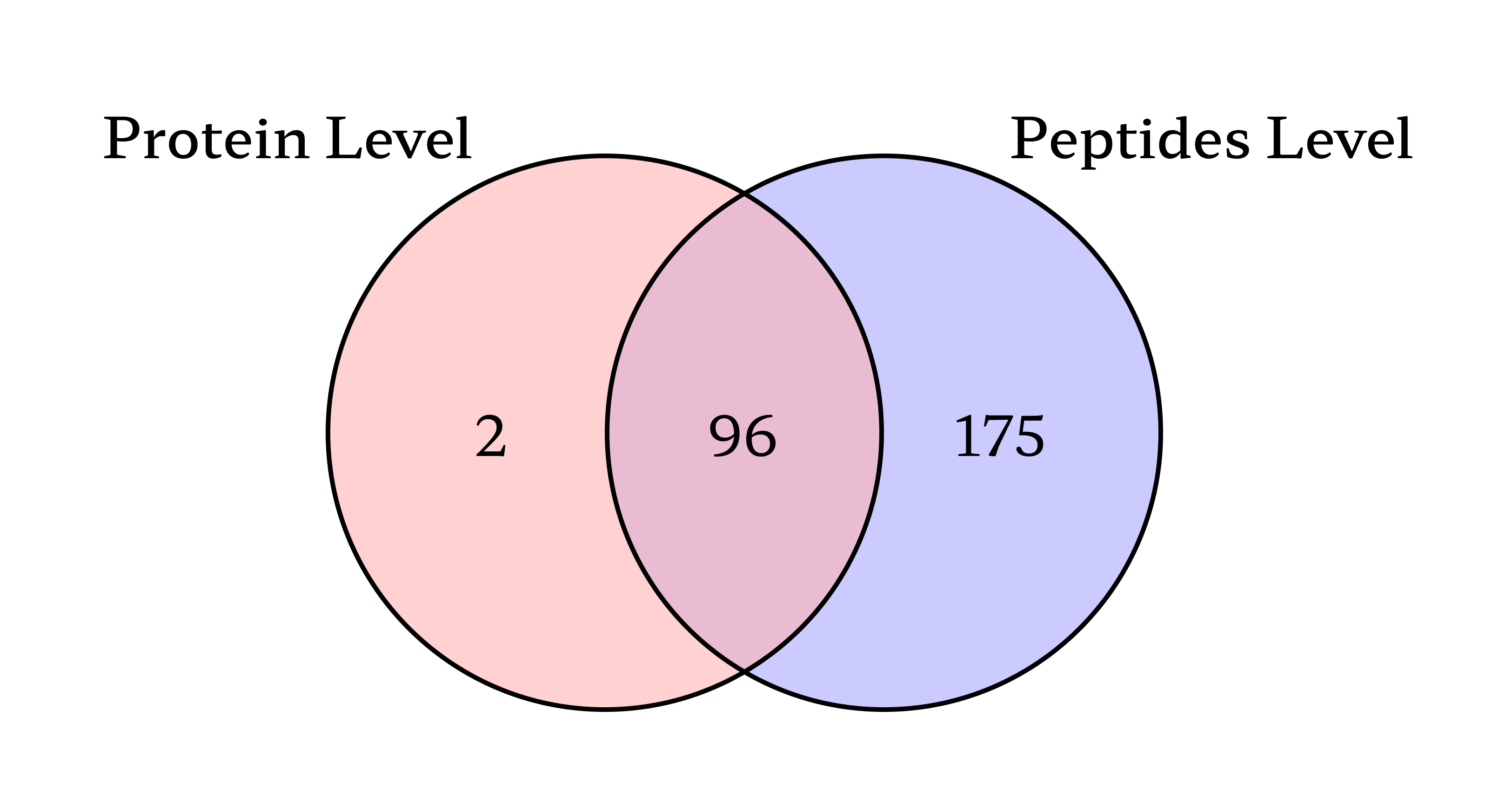

Supplement: btad076_Supplementary_Data [file btad076_supplementary_data.zip › Supplementary Figure 1-high.jpg]

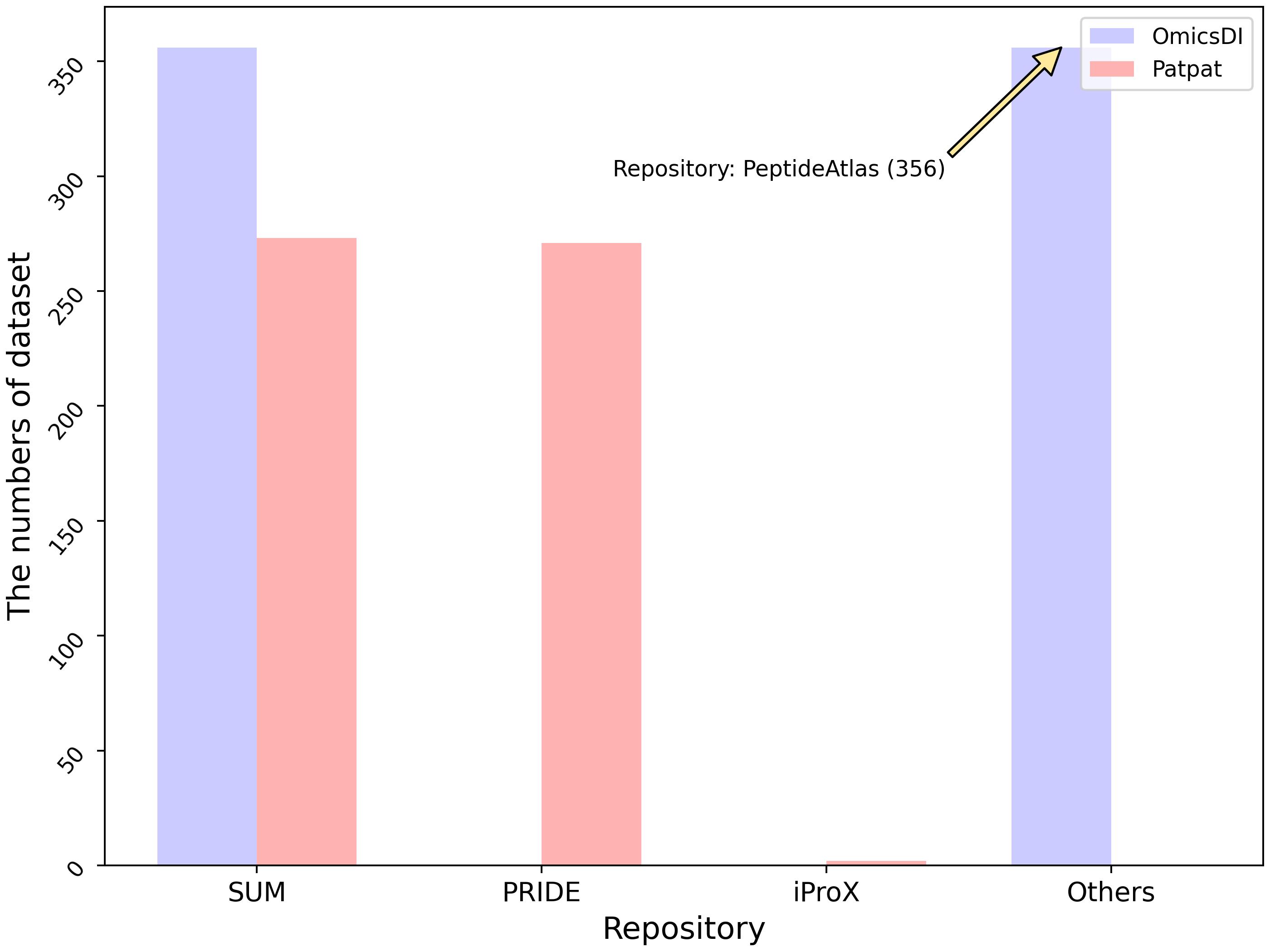

Supplement: btad076_Supplementary_Data [file btad076_supplementary_data.zip › Supplementary Figure 2-high.jpg]

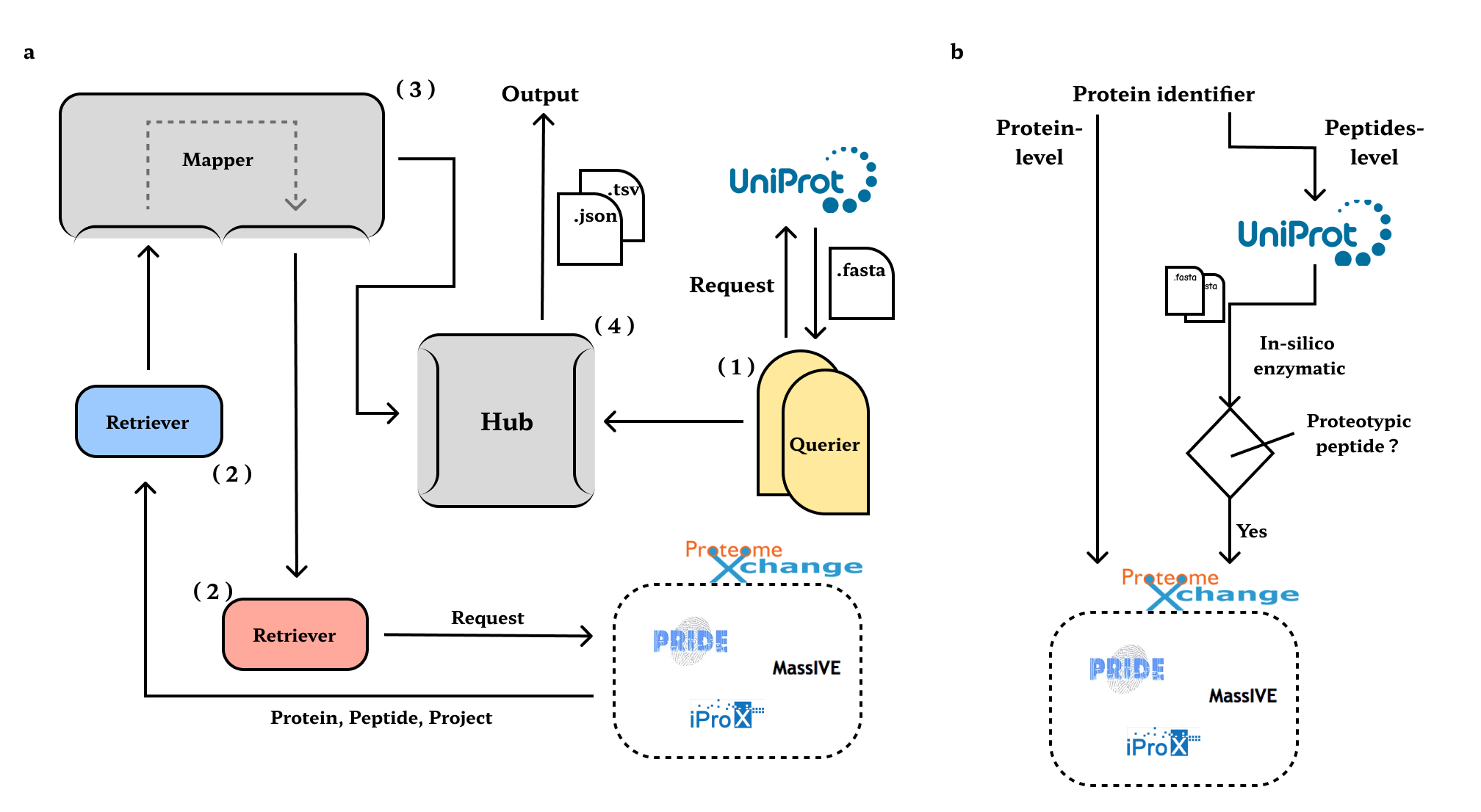

Supplement: btad076_Supplementary_Data [file btad076_supplementary_data.zip › 2022_Patpat Figure 1 V3-low.jpg]
